# Tubular Deficiency of Heterogeneous Nuclear Ribonucleoprotein F Elevates Systolic Blood Pressure and Induces Glycosuria in Mice

**DOI:** 10.1038/s41598-019-52323-1

**Published:** 2019-10-31

**Authors:** Chao-Sheng Lo, Kana N. Miyata, Shuiling Zhao, Anindya Ghosh, Shiao-Ying Chang, Isabelle Chenier, Janos G. Filep, Julie R. Ingelfinger, Shao-Ling Zhang, John S. D. Chan

**Affiliations:** 1 Université de Montréal, Département de Médecine Centre de recherche du Centre hospitalier de l’Université de Montréal (CRCHUM), Tour Viger-Pavillon R 900 Saint Denis Street Montreal, Quebec Canada, H2X 0A9 Montréal, Canada; 2 Université de Montréal Centre de recherche Maisonneuve-Rosemont Hospital 5415 boul. l’Assomption Montreal, Quebec Canada, H1T 2M4 Montréal, Canada; 3 Harvard Medical School Pediatric Nephrology Unit Massachusetts General Hospital 15 Parkman Street, WAC 709 Boston, Boston, MA 02114-3117 USA

**Keywords:** Molecular biology, Physiology, Systems biology, Zoology, Endocrinology, Nephrology

## Abstract

We reported previously that overexpression of heterogeneous nuclear ribonucleoprotein F (Hnrnpf) in renal proximal tubular cells (RPTCs) suppresses angiotensinogen (*Agt*) expression, and attenuates systemic hypertension and renal injury in diabetic *Hnrnpf*-transgenic (Tg) mice. We thus hypothesized that deletion of *Hnrnpf* in the renal proximal tubules (RPT) of mice would worsen systemic hypertension and kidney injury, perhaps revealing novel mechanism(s). Tubule-specific *Hnrnpf* knockout (KO) mice were generated by crossbreeding *Pax8-Cre* mice with floxed *Hnrnpf* mice on a C57BL/6 background. Both male and female KO mice exhibited elevated systolic blood pressure, increased urinary albumin/creatinine ratio, tubulo-interstitial fibrosis and glycosuria without changes in blood glucose or glomerular filtration rate compared with control littermates. However, glycosuria disappeared in male KO mice at the age of 12 weeks, while female KO mice had persistent glycosuria. Agt expression was elevated, whereas sodium-glucose co-transporter 2 (Sglt2) expression was down-regulated in RPTs of both male and female KO mice as compared to control littermates. *In vitro*, KO of *HNRNPF* in human RPTCs (HK-2) by CRISPR gRNA up-regulated *AGT* and down-regulated *SGLT2* expression. The Sglt2 inhibitor canagliflozin treatment had no effect on *Agt* and *Sglt2* expression in HK-2 and in RPTCs of wild-type mice but induced glycosuria. Our results demonstrate that *Hnrnpf* plays a role in the development of hypertension and glycosuria through modulation of renal *Agt* and *Sglt2* expression in mice, respectively.

## Introduction

The kidney contains all components of the renin-angiotensin system (RAS)^1–4^. Over-activation of the intrarenal RAS appears to be involved in various kidney diseases^5–7^. We and others have reported that overexpression of angiotensinogen (Agt, the sole precursor of all angiotensins) in RPTCs leads to systemic hypertension and kidney injury in transgenic (Tg) mice^8–10^, supporting the notion that enhanced intrarenal *Agt* expression and RAS activation play an important role in the development of hypertension and kidney injury.

Our lab has reported that heterogeneous nuclear ribonucleoprotein F (*Hnrnpf*) mediates insulin inhibition of *Agt* gene transcription through binding to the putative insulin-responsive element (*IRE*) in the rat *Agt* promoter^11,12^. We recently reported that overexpression of *Hnrnpf* in RPTCs suppresses *Agt* expression, and attenuates systemic hypertension and renal injury in male Akita (type 1 diabetic murine model) *Hnrnpf*-Tg mice^13^ and db/db (type 2 diabetic murine model) *Hnrnpf*-Tg mice^14^. Since sex differences may modulate the development of systolic blood pressure (SBP)^15,16^, we investigated whether *Hnrnpf* would affect intrarenal *Agt* expression in a sex-dependent manner. We generated tubule-specific *Hnrnpf* KO mice by employing the *Pax8-Cre/lox* system^17^ and monitored the development of phenotype in both male and female mice.

Here, we report that tubule-specific (Pax8) *Hnrnpf* KO leads to elevated SBP and kidney injury via up-regulation of *Agt* and down-regulation of *Sglt2* expression in RPTCs in both sexes and also results in glycosuria in a sex-dependent manner. KO of *HNRNPF* by CRISPR gRNA confirmed the up-regulation and down-regulation of *AGT* and *SGLT2* expression in human RPTCs (HK-2), respectively. Treatment with canagliflozin (an inhibitor of Sglt2) had no effect on *Agt* and *Sglt2* expression in HK-2 and in RPTCs of wild-type mice, whereas it induced glycosuria.

## Results

### Generation of tubular *Hnrnpf* KO Mice

**Figure 1:**
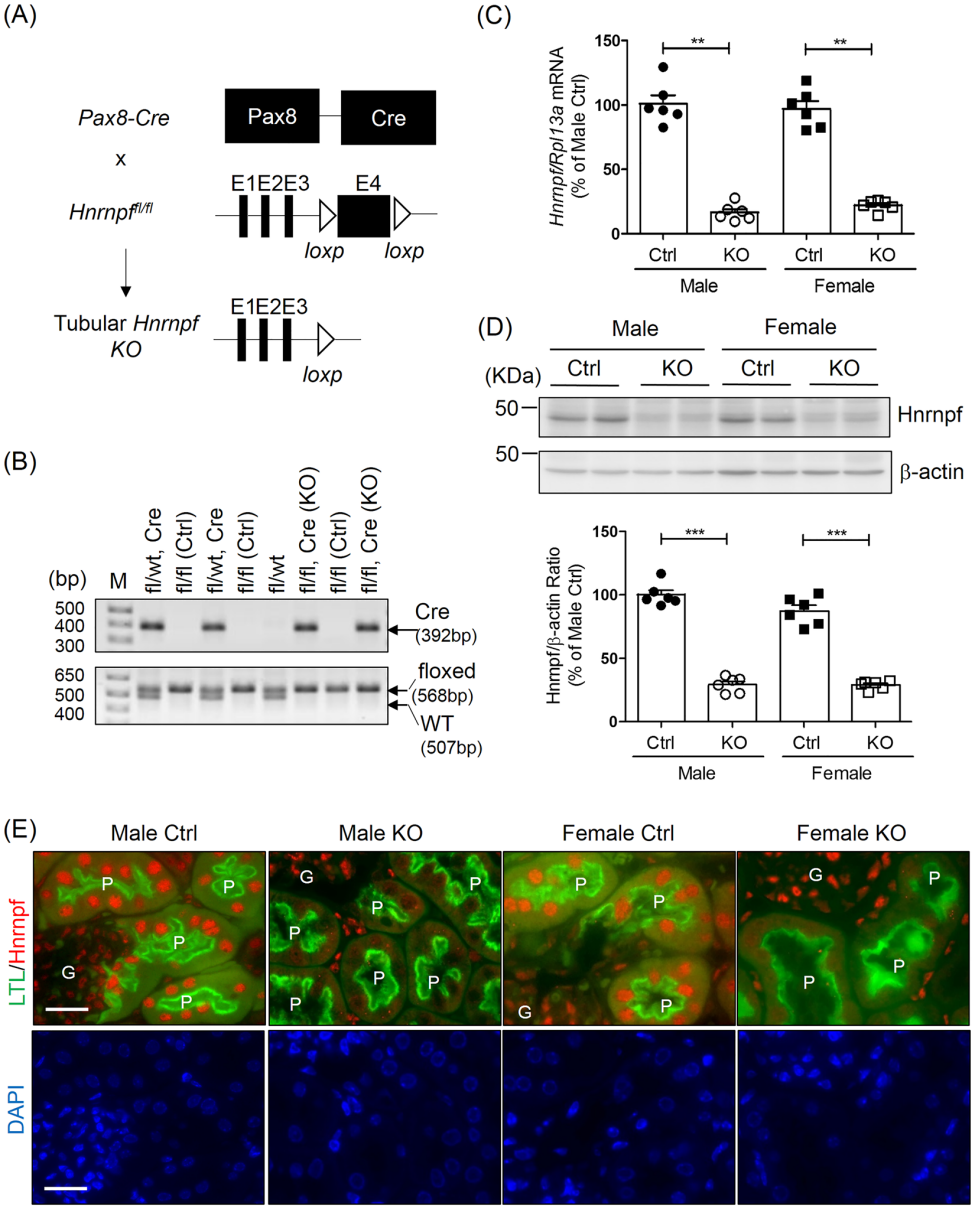
Generation of tubular *Hnrnpf* KO mice. (**A**) Schematic diagram describing the strategy of generating tubular *Hnrnpf* gene knockout mice. Exon 4 (E4) of the *Hnrnpf* gene is deleted; arrowheads: loxP sites. (**B**) Genotyping identification, the PCR bands of *Cre* (392 bp), *floxed* (568 bp) and *wild-type* (507 bp) alleles of *Hnrnpf* are indicated. Genotyping of representative litters are indicated; fl, *Hnrnpf* floxed; Control (Ctrl) (genotype: fl/fl) and KO (genotype: fl/fl, Cre). (**C**) Quantitative *Hnrnpf* mRNA expression level in male and female Ctrl and KO 24 week-old mice. **P < 0.01, KO versus Ctrl; n = 6 per group. (**D**) Representative WB and quantification of Hnrnpf protein expression in male and female Ctrl and KO 24 week-old mice. ***P < 0.005, KO versus Ctrl; n = 6 per group. (**E**) Immunostaining for Hnrnpf (red color) and a proximal tubular marker (lotus tetragonolobus lectin, LTL)(green color) in Ctrl and KO mice (original magnification ×600). DAPI staining (blue color) for cellular nucleus. Scale bars = 20 μm. G, glomerulus; P, proximal tubule.

Renal tubular *Hnrnpf* KO mice were generated by using *Pax8-Cre/lox* recombination strategy (Fig. 1A). *LoxP* sites were inserted to flank exon 4 of mouse *Hnrnpf* gene (Gene ID: 98758) which is localized on chromosome 6. Heterozygous of *Hnrnpf*-floxed allele mice were generated by cross-breeding male *Hnrnpf*-floxed mice with female *Pax8-Cre* mice. These mice were further crossbred to generate homozygous *Hnrnpf*-floxed allele and carried the *Cre* allele. PCR analysis of genomic DNA extracted from ear punch tissues to distinguish the genotype of *Cre* (392 bp), *floxed* (568 bp) and *WT* (507 bp) is shown in Fig. 1B. RT-qPCR revealed *Hnrnpf* mRNA expression in RPTs freshly isolated from male and female Ctrl and KO mice at the age of 8 weeks (Supplemental Fig. 1a) and 24 weeks (Fig. 1C). *Hnrnpf* mRNA was barely detectable in RPTs of both male and female KO mice at 8 and 24 weeks of age.

WB of isolated RPTs confirmed the expression of Hnrnpf at the age of 8 (Supplemental Fig. 1b) and 24 weeks (Fig. 1D) in Ctrl whereas Hnrnpf expression was significantly down-regulated in KO mice. No significant difference of Hnrnpf expression in RPTs was observed between male and female Ctrl as well as between male and female KO mice. Double immunofluorescence of kidney sections (Fig. 1E) with an anti-Hnrnpf antibody and LTL-FITC antibody, confirmed significantly higher Hnrnpf expression in RPTs from Ctrl than in KO mice.

### Physiological measurements in *Hnrnpf* KO mice

**Figure 2:**
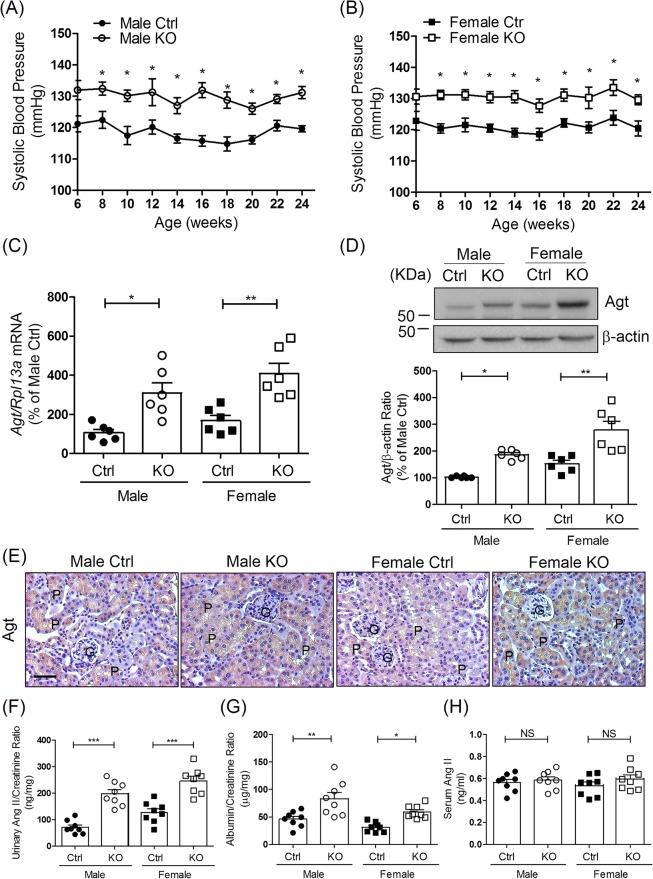
Systolic blood pressure (SBP) and intrarenal angiotensinogen (*Agt*) expression in tubular *Hnrnpf* KO mice. (**A**) Longitudinal average SBP measurement (performed two or three times per mouse per week in the morning without fasting) in (**A**) male and (**B**) female mice. Baseline SBP was measured daily over a 5-day period before initiation of actual measurement at week 6. Values are means ± SEM, n = 10 for each group. *P < 0.05, KO versus Ctrl. (**C**) *Agt* mRNA levels in male and female Ctrl and KO mice at the age of 24 weeks. *P < 0.05, **P < 0.01, n = 6 per group, KO versus Ctrl. (**D**) Representative WB of Agt protein expression and quantitation of Agt expression in Ctrl and KO groups from 24 week-old male and female mice. *P < 0.05, **P < 0.01, n = 6 per group, KO versus Ctrl. (**E**) Representative immunostaining for Agt in Ctrl and KO mice (original magnification ×200). Scale bars = 50 μm. G, Glomerulus; P, proximal tubule. (**F**) Urinary Ang II, (**G**) ACR and (**H**) serum Ang II levels at week 24 in Ctrl and KO mice. Urinary Ang II and albumin levels were normalized with urinary creatinine levels. Values are mean ± SEM, n = 8 per group. *P < 0.05, **P < 0.01 and ***P < 0.005; KO versus Ctrl.

Deletion of renal tubular *Hnrnpf* did not influence body weight gain nor the non-fasting blood glucose levels in both male and female mice from the age of 6 to 24 weeks (Supplemental Fig. 1c–f, respectively). Longitudinal SBP measurements revealed consistently higher SBP in both male (Fig. 2A) and female (Fig. 2B) KO mice aged week 6 to 24 compared to Ctrl. Significant increases of *Agt* mRNA and protein expression were detected in both male and female KO mice compared to Ctrl at 8 weeks (Supplemental Fig. 2a) and 24 weeks of age (Fig. 2C,D, respectively). No significant difference of Agt expression in RPTs was observed between male and female Ctrl as well as between male and female KO mice. These were confirmed with immunostaining (Fig. 2E).

Increased urinary Ang II and urinary albumin/creatinine ratio (ACR) were also observed in both male and female KO mice compared to Ctrl at 24 weeks of age with no significant difference between male and female Ctrl as well as between male and female KO mice. (Fig. 2F,G, respectively). In contrast, body weight (BW), kidney weight (KW)/BW, GFR and glomerular tuft volume did not differ significantly between KO mice and Ctrl at 24 weeks of age (Table 1). Twenty-four h urine volume were significantly increased but not food and water intake in both male and female KO mice as compared to Ctrl. No differences were detected in serum and urine levels of sodium, calcium and phosphorus between male and female Ctrl and KO mice. We detected no significant differences in serum Ang II among different groups of mice (Fig. 2H).

**Table 1:** Physiological parameters of mice at 24 weeks of age.

	Male			Female		
	Ctrl	*Hnrnpf* KO	*p*	Ctrl	*Hnrnpf* KO	*p*
Body weight (g)	34.35 ± 0.96	34.94 ± 0.68	NS	24.81 ± 0.89	23.55 ± 0.86	NS
KW/BW (mg/g)	9.35 ± 0.58	8.38 ± 0.41	NS	9.97 ± 0.29	9.05 ± 0.45	NS
GFR(µL/min)/BW(g)	7.5 ± 0.45	7.7 ± 0.57	NS	7.9 ± 0.64	6.8 ± 0.34	NS
Glomerular tuft volume (10^3^ µm^3^)	117.3 ± 8.92	128.2 ± 10.94	NS	120.2 ± 9.90	129.5 ± 5.88	NS
Urine volume (µl/24 h)	871.7 ± 36.1	1198.0 ± 102.7	*	265.0 ± 63.3	505.8 ± 41.5	**
Food intake (mg/24 h)	333.3 ± 42.2	333.3 ± 33.3	NS	566.7 ± 76.0	516.7 ± 54.3	NS
Water intake (ml/24 h)	1.97 ± 0.08	2.37 ± 0.24	NS	2.30 ± 0.29	2.55 ± 0.13	NS
Serum Na (mmol/L)	149.7 ± 2.3	151.5 ± 1.4	NS	147.6 ± 2.1 ^#^	148.7 ± 1.4	NS
Serum Ca (mmol/L)	2.22 ± 0.03	2.21 ± 0.04	NS	2.34 ± 0.11 ^#^	2.20 ± 0.03	NS
Serum P (mmol/L)	2.89 ± 0.20	2.95 ± 0.29	NS	3.37 ± 0.36	3.13 ± 0.14	NS
Urine Na/Cr (mmol/g Cr)	741.0 ± 73.1	709.8 ± 25.0	NS	398.5 ± 61.1	353.2 ± 32.9	NS
Urine P/Cr (mmol/g Cr)	107.2 ± 17.2	101.2 ± 17.9	NS	73.3 ± 10.9	79.7 ± 12.6	NS

Values are mean ± SEM; n = 6/group.

KW/BW. Kidney Weight/Body Weight; Na. sodium; Ca. calcium; P. phosphorus; Cr. creatinine.

**p < 0.01, *p < 0.05, NS, not significant.

### Tubulo-interstitial fibrosis in *Hnrnpf* KO mice

**Figure 3:**
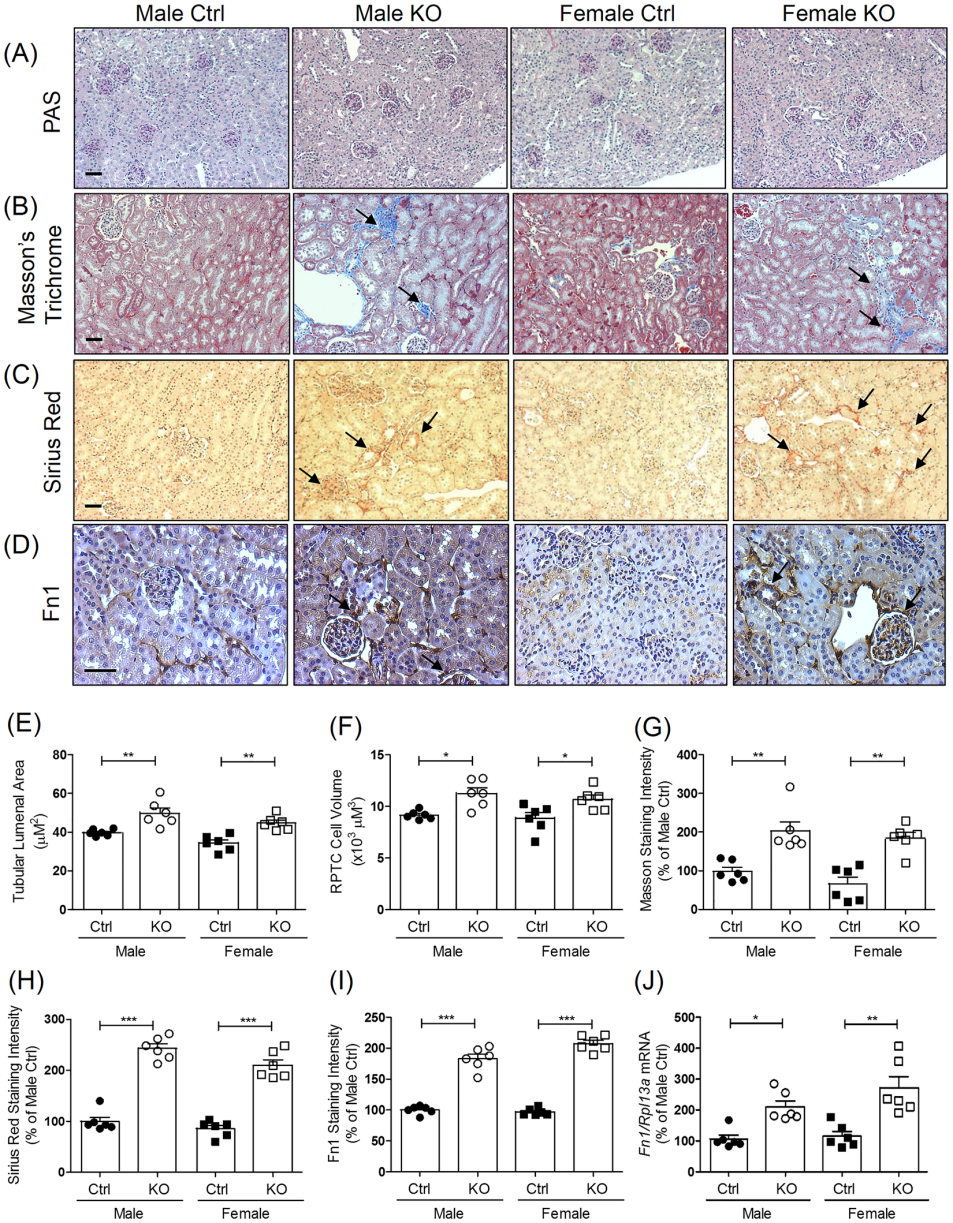
Tubulo-interstitial fibrosis in mouse kidneys. (**A**) Representative image of Periodic acid-Schiff (PAS) staining, (**B**) Masson’s trichrome staining, (**C**) Sirius Red staining and (**D**) fibronectin-1 (Fn1) immunostaining (original magnification ×100) in kidneys from male and female Ctrl and KO mice at the age of 24 weeks. (**G**) Glomerulus; P, proximal tubule. Scale bars = 50 μm. Semi-quantitation of tubule lumenal area (**E**), RPTC volume (**F**), Masson’s trichrome staining (**G**), Sirius Red staining (**H**) and Fn1 immunostaining (**I**) of Ctrl and KO mice at the age of 24 weeks. RT-qPCR of *Fn1*(J) in freshly RPTs from male and female Ctrl and KO mice. Values are means ± SEM, n = 6. *P < 0.05, **P < 0.01, ***P < 0.005; KO versus Ctrl.

PAS staining of kidney sections showed no obvious structural changes in KO mice at the age of 24 weeks (Fig. 3A). Increased fibrosis on Masson’s Trichrome staining (Fig. 3B) and increased expression of collagen on Sirius Red staining (Fig. 3C), and fibronectin 1 (Fn1) immunostaining (Fig. 3D) was, however, noted in glomerulo-tubular regions in KO mice as compared to Ctrl at the age of 24 weeks. Semi-quantification of tubular lumenal area (Fig. 3E), RPTC volume (Fig. 3F), Masson’s Trichrome staining (Fig. 3G), Sirius Red staining (Fig. 3H) and Fn1 immunostaining (Fig. 3I) revealed an increase of tubular lumenal area, RPTC volume, Masson’s Trichrome and Sirius Red staining and Fn1 immunostaining in KO mice as compared to Ctrl, respectively. These findings were associated with significant increases of mRNA expression of *Fn1* (Fig. 3J) by RT-qPCR in isolated RPTs of KO mice as compared with Ctrl.

### Glycosuria and Sglt2 expression in *Hnrnpf* KO mice

**Figure 4:**
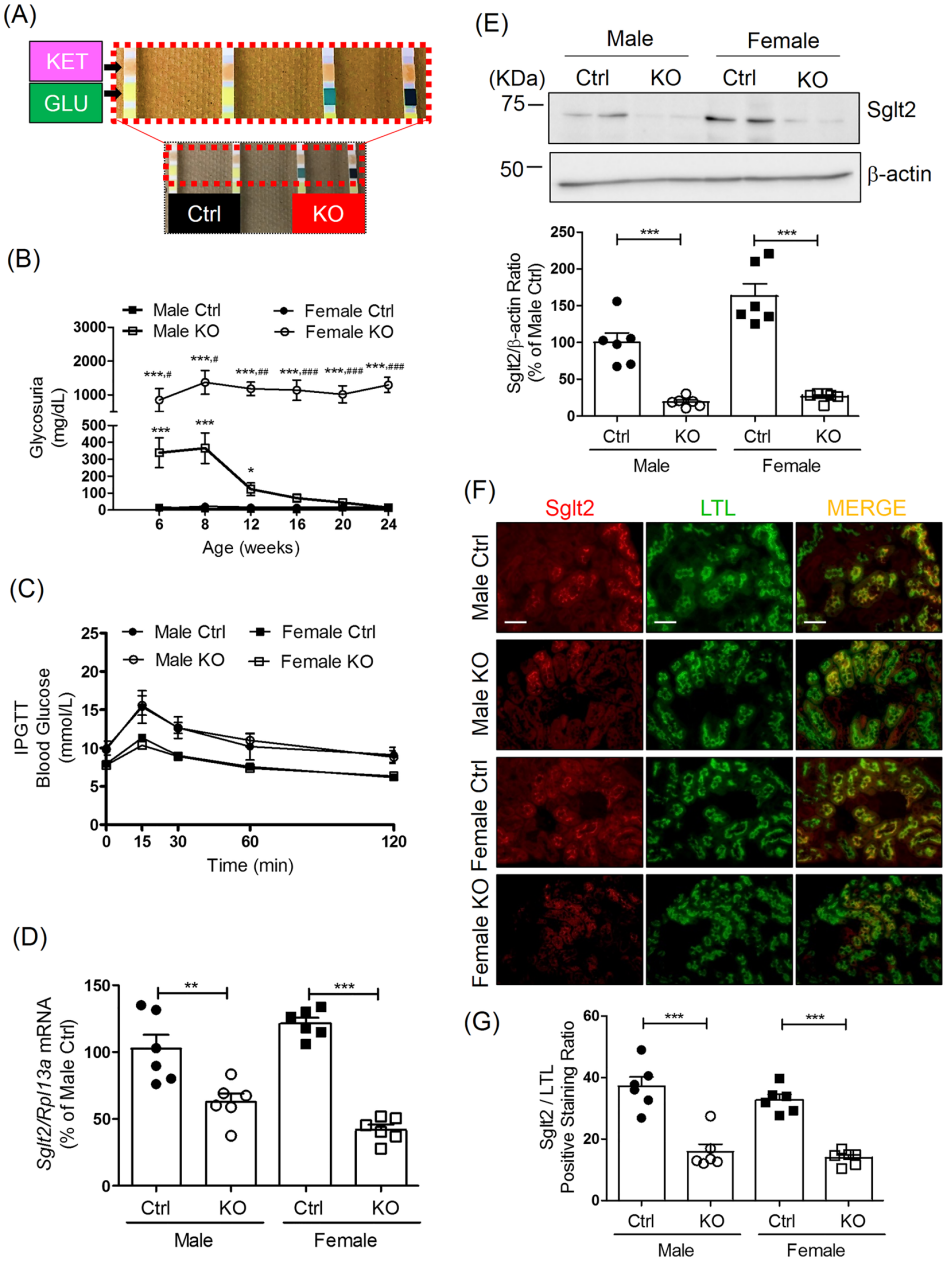
Glycosuria and Sglt2 expression in *Hnrnpf* KO mice. (**A**) Urinary glucose in Ctrl and KO mice detected by dipstick test at the age of 6 weeks. (**B**) Longitudinal urinary glucose levels in male and female KO mice and Ctrl from the age of 6 weeks to 24 weeks measured by glucose colorimetric kit. Values are means ± SEM, n = 6. **P < 0.01; ***P < 0.005. KO versus Ctrl. ^##^P < 0.01; ^###^P < 0.005^,^ female KO versus male KO. IPGTT test in male and female (**C**) Ctrl and KO mice at the age of 23 weeks. (**D**) Ratio of *Sglt2*/*Rpl13a* mRNA expression quantified by RT-qPCR and (**E**) Representative WB of Sglt2 protein expression in male and female mouse RPTs at the age of 24 weeks. Values are means ± SEM, n = 6. ***P < 0.005; KO versus Ctrl. (**F**) Double immunostaining of Sglt2 and LTL (magnification x100) and semi-quantification of Sglt2/LTL immunostaining ratio (**G**) in male and female Ctrl and KO mouse kidneys at the age of 24 weeks. Values are Sglt2/LTL positive staining ratio, n = 6. ***P < 0.005, KO versus Ctrl.

Unexpectedly, increased glucose excretion was detected in the urine using dipsticks in both male and female KO mice at age 6 weeks (Fig. 4A). From 8 weeks of age, urinary glucose levels in male KO mice steadily decreased and returned to levels similar to Ctrl mice at 12 weeks of age (Fig. 4B). In contrast, urinary glucose excretion steadily increased from week 6 in female KO mice, reached an apparent plateau at the age of 12 weeks and did not abate (Fig. 4B). No changes in urinary glucose level were detected in male and female Ctrl.

Having observed that both male and female *Hnrnpf* KO mice develop glycosuria, we performed intraperitoneal glucose tolerance test (IPGTT) at the age of 23 weeks in male and female mice (Fig. 4C). KO of *Hnrnpf* in RPTs did not influence the glucose tolerance in either male or female KO mice.

RT-qPCR revealed lower *Sglt2* expression in RPTs isolated from both male and female KO mice at 8 weeks of age (Supplemental Fig. 2b) and 24 weeks of age (Fig. 4D) as compared with Ctrl. At both 8 and 24 weeks of age, *Sglt2* expression decreased by ~40% in RPTs of male *Hnrnpf* KO mice as compared to Ctrl, whereas persistently lower *Sglt2* expression (decreased by ~60% of baseline level) was observed in RPTs from female KO mice. However, no significant difference of *Sglt2* expression in RPTs was observed between male and female Ctrl as well as between male and female KO mice. WB of isolated RPTs confirmed these changes at the protein level (Fig. 4E). Consistently, semi-quantitation of immunofluorescence staining with anti-Sglt2 antibodies and LTL-FITC confirmed reduced Sglt2 expression in RPTs of 24 week-old KO mice as compared to Ctrl (Fig. 4F,G, respectively). No significant changes were detectable in *Slc5a1* (*Sglt1*) mRNA expression in RPTs isolated from both male and female KO mice as compared to Ctrl (Supplemental Fig. 2c).

### Effect of canagliflozin treatment on Agt and Sglt2 expression in RPTCs *in vivo*

**Figure 5:**
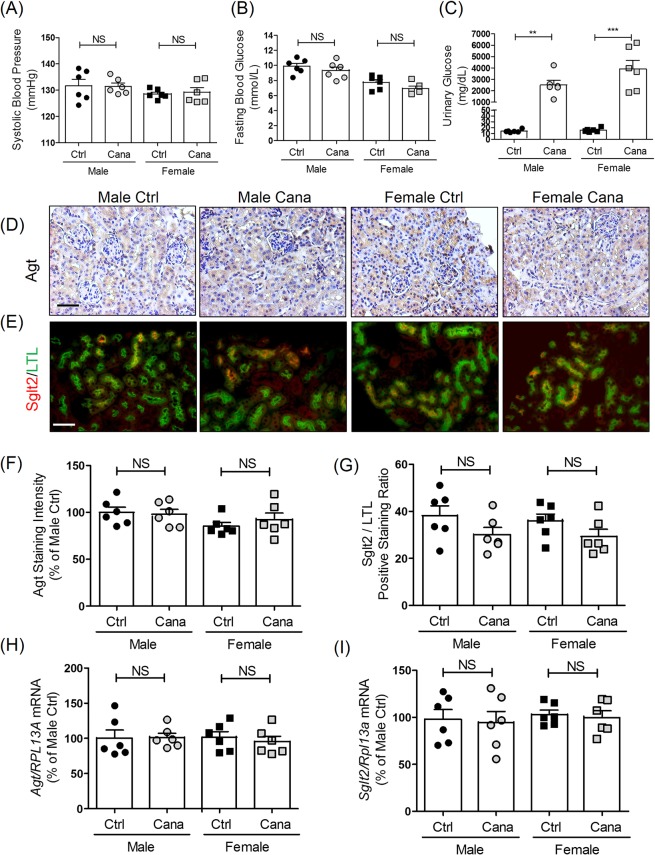
Effect of canagliflozin treatment on blood and urinary glucose levels, AGT and SGLT2 expression in mice. (**A**) SBP, (**B**) blood glucose and (**C**) urinary glucose levels after 4 weeks of treatment with or without canagliflozin in adult male and female wild-type (WT) mice. Values are means ± SEM, n = 6. **P < 0.01, ***P < 0.005; canagliflozin-treated versus non-treated mice. Immunostaining for Agt (**D**) and immunoflourescent staining of Sglt2 (**E**) in the kidneys of WT male and female mice with or without 4 weeks of canagliflozin treatment. Magnification x 200. Scale bars = 50 μm. Semi-quantification of Agt (**F**) and Sglt2 (**G**) immunostaining in male and female WT mouse kidneys after 4 weeks of treatment with or without canagliflozin. RT-qPCR of *Agt* (**H**) and *Sglt2* (**I**) expression in isolated RPTs of WT male and female mice with or without 4 weeks of canagliflozin treatment. Values are means ± SEM, n = 6. NS, not significant; canagliflozin treated versus non-treated mice.

To investigate the role of Sglt2 on Agt and Sglt2 expression in RPTCs *in vivo*, wild-type mice were treated with the selective Sglt2 inhibitor canagliflozin (0.2 mg/ml in drinking water). Four weeks of canagliflozin treatment had no detectable effects on SBP (Fig. 5A) and blood glucose levels (Fig. 5B) in either male or female mice but enhanced the development of glycosuria in both male and female mice (Fig. 5C) as compared to non-treated mice. Immunostaining for Agt (Fig. 5D) and immunofluorescent staining for Sglt2 (Fig. 5E) revealed that canagliflozin treatment had no effect on Agt expression or Sglt2 expression in RPTCs of either male or female mice. These observations were confirmed by semi-quantitation of Agt (Fig. 5F) and Sglt2 (Fig. 5G) expression and qPCR of *Agt* and *Sglt2* mRNA expression in isolated RPTs (Fig. 5H,I, respectively).

### *AGT* and *SGLT2* expression in HK-2 with or without *HNRNPF* KO

**Figure 6:**
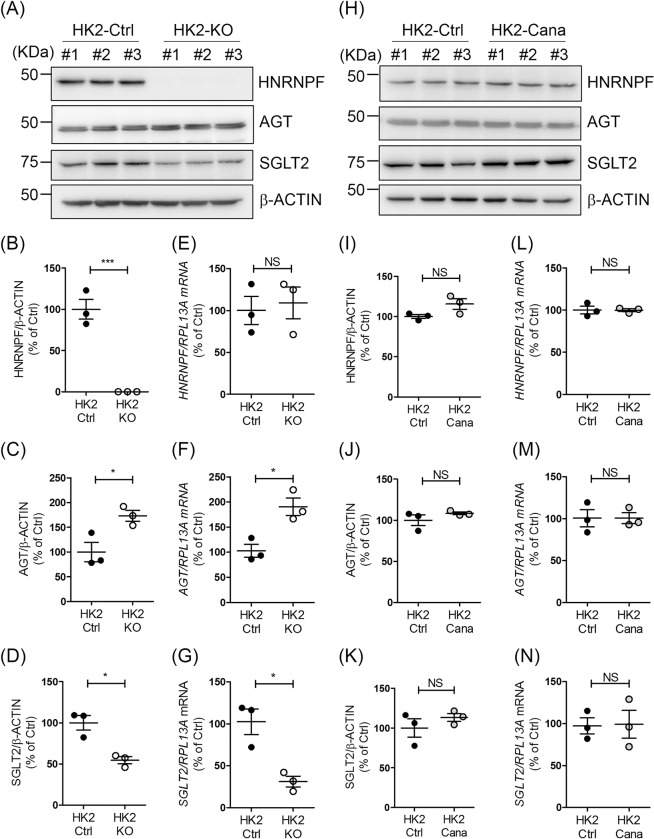
*AGT* and *SGLT2* expression in HK-2 with or without *HNRNPF* KO. (**A**) WB, (**B**–**D**) semi-quantitation of WB and (**E**–**G**) RT-qPCR of HNRNPF, AGT, SGLT2 and β-ACTIN in different clones of HK-2 Ctrl and HK-2 with *HNRNPF* KO by CRISPR gRNA. Values are means ± SEM, n = 3. *P < 0.05, **P < 0.01; HK-2-HNRNPF KO versus HK-2 Ctrl. (**H**) WB, (**I**–**K**) semi-quantitation of WB and (L)(M)(N) RT-qPCR of HNRNPF, AGT, SGLT2 and β-ACTIN of expression in HK-2 with or without canagliflozin (Cana) (0.5 mM) treatment for 24 hours. Values are means ± SEM, n = 3. NS, not significant. HK-2-Cana versus HK-2 Ctrl.

To validate our *in vivo* observations, we generated HK-2 cells with *HNRNPF* KO by CRISPR gRNA technology. Consistent with our *in vivo* observation, immunoblots revealed that HK-2 cells with *HNRNPF* KO exhibited non-detectable HNRNPF (Fig. 6A,B), higher AGT (Fig. 6A,C) and lower SGLT2 protein expression (Fig. 6A,D) as compared to control HK-2. These findings were confirmed by RT-qPCR of *HNRNPF* (Fig. 6E), *AGT* (Fig. 6F) and *SGLT2* expression (Fig. 6G), respectively. Finally, in human cells expression of HNRNPF (Fig. 6H,I), AGT (Fig. 6H,J) and SGLT2 protein (Fig. 6H,K) and mRNA (Fig. 6L–N, respectively) did not differ significantly in HK-2 cells treated with canagliflozin and untreated cells, indicating a lack of causality of inhibition of SGLT2 activity and *AGT* and *SGLT2* expression in RPTCs.

## Discussion

Our results identify a novel mechanism by which Hnrnpf affects the development of hypertension and glycosuria in mice through modulation of intrarenal *Agt* and *Sglt2* expression, respectively.

Hnrnpf, a member of the 30 pre-mRNA-binding protein family, modulates gene expression at both transcriptional and post-transcriptional levels^11–14,18–21^. Hnrnpf engages in alternative splicing of various genes and associates with TATA-binding protein, RNA polymerase II, nuclear cap-binding protein complex and various transcription factors to modulate gene expression^22^. We have reported previously that Hnrnpf overexpression in RPTCs attenuates hypertension and kidney injury in both diabetic Akita^13^ and db/db^14^ mice via inhibition of intrarenal Agt expression, implying an important role for Hnrnpf in modulating the development of hypertension and nephropathy in diabetic mice.

Our present findings document that genetic deletion of *Hnrnpf* in tubules enhances renal *Agt* expression, hypertension development and kidney injury in both non-diabetic male and female mice. These observations are consistent with our hypothesis that Hnrnpf plays an important role in the development of hypertension and tubulo-interstitial fibrosis via modulation of *Agt* and pro-fibrotic genes expression in RPTs.

Initially, we generated global *Hnrnpf* KO mice by cross-breeding a general Cre-deleter mouse line (CMV-Cre; B6.C-Tg(CMV-cre)1Cgn/J) with our *Hnrnpf*^fl/fl^ mice on a C57BL/6 background to explore the phenotype of global *Hnrnpf* KO mice and found that like the global *Hnrnpk* deletion^23^, global *Hnrnpf* KO also results in embryonic death (Supplemental Fig. 3). To circumvent this issue, we generated renal tubule-specific *Hnrnpf* KO mice by cross-breeding our *Hnrnpf*^fl/fl^ mice with a renal tubule-specific Cre deleter (Pax8-Cre; B6.129P2(Cg)-*Pax8*^*tm1*.*1*(*cre*)*Mbu*^/J) mouse line^17^. Several labs have also successfully employed Pax8-Cre mice to delete genes in renal tubules^24–26^. Our homozygous Pax8-*Hnrnpf* KO mice are viable and fertile without symptoms of body weight loss, physiological imbalance and altered hearing. However, they develop hypertension and elevated ACR with increased Agt expression in RPTCs by 8 weeks of age (Supplemental Fig. 4a,b). Since Pax8 is also expressed in the thyroid gland and hindbrain^17^, it is possible that Pax8-*Hnrnpf* KO mice might exhibit abnormality in thyroid gland and hindbrain thereby indirectly affecting cardiac and renal function. However, we did not detect significant changes in *Hnrnpf* mRNA levels or serum T_4_ levels in Pax8-*Hnrnpf* KO mice (Supplemental Fig. 5) or histological changes in thyroid gland and hindbrain. Thus, our Pax8-*Hnrnpf* KO mouse appears to be a valid murine model with which to study the phenotype with tubule-specific *Hnrnpf* KO.

An unexpected finding of our present study was that *Hnrnpf* deletion led to glycosuria with reduced expression of *Sglt2* in RPTs of *Hnrnpf* KO mice. Intriguingly, serum and urine levels of Na, Ca and P did not differ between *Hnrnpf* KO mice and Ctrl. The phenotype of glycosuria appears to be similar to that reported in *Sglt2* deficient mice^27^ and in patients with familial renal glycosuria (FRG)^28–30^ but differs from that in Sweet Pee mice, which are characterized by elevated urinary excretion of calcium and magnesium and growth retardation^31^, as well as from that in patients with renal Fanconi syndrome^32^. Intriguingly, male *Hnrnpf* KO mice exhibited a transient glycosuria between the ages of 6 and 12 weeks and then returned to levels similar to Ctrl. Furthermore, glycosuria correlates with reduced *Sglt2* expression in RPTs of male *Hnrnpf* KO mice. In contrast, female *Hnrnpf* KO mice displayed persistent glycosuria throughout 6 to 24 weeks of age with similar inhibition of *Sglt2* expression observed at both 8 and 24 weeks of age. These data would indicate that male sex hormones rather than female sex hormones may modulate *Sglt2* expression in *Hnrnpf* KO mice. Indeed, this notion had been suggested by Sabolic’s group^33,34^ who implicated androgen, but not estradiol up-regulates Sglt2 expression and activity in mice.

To explore the impact of Sglt2 inhibition on *Agt* expression, we treated HK-2 cells and WT mice with canagliflozin. Canagliflozin had no detectable effects on SBP and blood glucose levels but enhanced the development of glycosuria in both male and female mice as compared to non-treated mice. Furthermore, canagliflozin treatment had no effect on the expression of *Agt* and *Sglt2* expression in RPTs of mice. Thus, our data would argue against a causal relationship between Sglt2 inhibition and *Agt* expression in RPTCs; rather our data would indicate that inhibition of *Hnrnpf* expression modulates both *Agt* and *Sglt2* expression in RPTCs.

Finally, to replicate our *in vivo* observations, we studied a human renal proximal tubular cell line (HK-2)^35^. By employing CRISPR gRNA technology, we obtained several clones of HK-2 cells with *HNRNPF* KO. Consistent with our findings in *Hnrnpf* KO mice, HK-2 with *HNRNPF* KO displayed significantly higher *AGT* and lower *SGLT2* expression as compared to HK-2 controls. These data lend further support our previous observations that Hnrnpf down-regulates RPT Agt expression and RAS activation, leading to improve tubulo-interstitial fibrosis in the kidney. Moreover, consistent with our *in vivo* results, canagliflozin treatment had no effect on SGLT2 and AGT expression in HK-2 cells.

At present, the underlying mechanism(s) by which genetic deletion of *HNRNPF* led to down-regulation of *SGLT2* transcription in HK-2 cells are unclear. One possibility might be that HNRNPF affects *SGLT2* transcription at the promoter activity level. This is unlikely since transfection of the *HNRNPF* cDNA did not affect the *SGLT2* promoter activity (pGL4.2/*SGLT2*-N-1,986/+22 promoter) in HK-2 cells (Supplemental Fig. 6a). However, we could not rule out the possibility of putative HNRNPF-*response element*(*s*) upstream of 2 kb of the *SGLT2* promoter. The second possibility is that *HNRNPF* deletion might alter the splicing of *SGLT2* to yield mutant forms of SGLT2. This is also unlikely since only one species of *SGLT2* was detectable in HK-2 cells with *HNRNPF* KO, which was similar to the size of *SGLT2* in HK-2 (Supplemental Fig. 6b). The third possibility is that HNRNPF might affect *SGLT2* mRNA stability. This notion is supported by the observations of Chu *et al*.^19^ that HNRNPF regulates YAP expression via binding to the 3′UTR of *YAP* to affect its mRNA stability and of Decorsiere *et al*.^21^ that Hnrnph/f interacts with a G-quadruplex in maintaining p53 pre-mRNA 3′-end processing during DNA damage. The fourth possibility is that deletion of *HNRNPF* might suppress other un-defined signaling pathway(s) or factor(s) that might have a greater impact (stimulation) on *SGLT2* expression and activity. Clearly, further studies are needed to elucidate the mechanisms underlying HNRNPF down-regulation of *SGLT2* expression.

The exact mechanism(s) of Hnrnpf regulation of *Agt* expression is unknown. One possibility is that Hnrnpf binds to the *insulin-responsive element* (*IRE*) in the *Agt* promoter^11,12^ and functions as a negative trans-acting protein to inhibit the binding of other positive trans-acting factor(s) to TATA-binding protein (TBP) and RNA polymerase II, subsequently attenuating *Agt* transcription. This possibility is supported by the studies of Yoshida *et al*.^22^ showing that Hnrnpf is associated with TBP, RNA polymerase II and nuclear cap-binding protein complex. A second possibility is that Hnrnpf is associated with Hnrnpk to form an Hnrnpf/k complex and that the Hnrnpf/k complex is more effective in inhibiting *Agt* transcription. Indeed, we have previously reported that Hnrnpf co-immunoprecipitated with Hnrnpk and that co-transfection of Hnrnpf with HnrnpK was more effective in inhibiting *Agt* transcription than either Hnrnpf or HnrnpK alone^35^. A third possibility is that the Hnrnpf/k complex might recruit unidentified repressor molecules and subsequently repress *Agt* transcription. This third possibility is supported by the studies of Denisenko *et al*.^36^ demonstrating that Hnrnpk could bind the murine repressor Zik1. Clearly, more work is needed to elucidate the precise molecular mechanism of action of Hnrnpf on *Agt* transcription in RPTCs.

In summary, the present study reveals a novel role for Hnrnpf in the development of hypertension, tubule-interstitial fibrosis and glycosuria in mice via up-regulation of *Agt* and down-regulation of *Sglt2* expression in RPTs, respectively. With the recent development of SGLT2 inhibitors as a novel treatment for diabetic patients^37–41^, it would be important to understand the regulation of *SGLT2* expression at the molecular level. Our findings raise the possibility that *Hnrnpf* KO mice may be a useful animal model for advancing studies on *SGLT2* regulation and familial renal glycosuria in human.

## Methods

### Chemical and reagents

Fluorescein isothiocyanate-labeled inulin and canagliflozin (Invokana) were purchased from Sigma-Aldrich (Oakville, ON, Canada) and Janssen Inc. (Toronto, ON, Canada), respectively. Dulbecco Modified Eagle Medium (DMEM) (Cat. No. 11966-025), Ham’s F12 medium (Cat. No. 11765-054) and fetal bovine serum (FBS) were bought from Gibco (Thermo Fisher Scientific, Montreal, QC, Canada). Oligonucleotides were synthesized by Integrated DNA Technologies, Inc. (Coralville, IA) and listed in Supplemental Table 1. Restriction and modifying enzymes were purchased from New England Biolabs (Whitby, ON, Canada). The sources of antibodies used are listed in Supplemental Table 2. HK-2 (an immortalized human renal proximal tubular cell line) (Cat. No. CRL-2190) was obtained from American Tissue Cell Collection (ATCC) (Manassas, VA) (http://www.atcc.org). Human *SGLT2* gene promoter (N-1,986/+22) was amplified from HK-2 genomic DNA by PCR with specific primers (Supplemental Table 1) and then inserted into pGL4.20 reporter vector (Promega, Sunnyvale, CA) at *Xho1*and *Bgl II* restriction sites.

### Generation of tubular *Hnrnpf* KO mice

Tubule-specific *Hnrnpf* KO mice were generated by cross-breeding male *Hnrnpf*-floxed mice with female *Pax8-Cre* mice^17^ (Stock number: 028196; Jackson Laboratory, Bar Harbor, ME). Briefly, the mouse *Hnrnpf* gene (Gene ID: 98758) is localized on chromosome 6: 117,900,340-117,925,622. Four exons have been identified in *Hnrnpf* with the ATG start codon and TAG stop codon both located in exon 4. The lox-modified *Hnrnpf* targeting vector was created by including 5′ and 3′ homology arms as well as two *loxP* sites flanking the fourth exon region amplified from SV129 BAC genomic DNA and confirmed by sequencing. C57BL/6 ES cells were used for gene targeting (Cyagen Biosciences, Santa Clara, CA). These mice allow the excision of exon 4 of *Hnrnpf* gene and disruption of the protein expression in the presence of Cre recombinase. By cross-breeding male *Hnrnpf*-floxed mice with female *Pax8-Cre* mice, heterozygous *Hnrnpf*-floxed allele mice were generated (genotype: *Hnrnpf*^*fl/wt*^,*Cre*). These mice were back-crossbred to generate homozygous *Hnrnpf*-floxed allele and carrying the *Cre* allele (genotype: *Hnrnpf*^*fl/fl*^,*Cre*). The Pax8-*Hnrnpf* KO mice (genotype: *Hnrnpf*^*fl/fl*^,*Cre*) and control littermates (Ctrl) (genotype: *Hnrnpf*^*fl/fl*^) as well as heterozygous littermates (genotype: *Hnrnpf*^*fl/wt*^,*Cre* and *Hnrnpf*^*fl/wt*^) were used in the present studies. Experimental mice were generated from at least three different breeding couples. Offspring were genotyped by PCR to detect the *Cre*-recombinase as well as the presence or absence of the 5′ *loxP* site using specific primers (Supplemental Table 1).

### Physiological studies

Age- and sex-matched male and female KO (genotype: *Hnrnpf*^*fl/fl*^*:Cre*) and control littermates (Ctrl) (genotype: *Hnrnpf*^*fl/fl*^) were studied. Animal care and procedures followed the Principles of Laboratory Animal Care (NIH Publication No. 85-23, revised 1985 (http://grants1.nih.gov/grants/olaw/references/phspol.htm) and were approved by the CRCHUM Animal Care Committee.

Weekly random blood glucose levels were measured in mice by Accu-Chek Performa (Roche Diagnostics, Laval, QC, Canada). SBP was measured with BP-2000 tail-cuff (Visitech Systems, Apex, NC) at least 2 to 3 times per week per animal in the morning without fasting as previously described^13,14^. Baseline SBP was measured daily over a 5-day period before initiation of actual measurement at 6 weeks of age.

At 24 weeks of age, twenty-four h prior to euthanasia, mice were housed individually in metabolic cages. Food, water consumption, and urine output were recorded. Mouse serum and urine samples were extracted with C18 Sep-Pak columns (Waters, Mississauga, ON) and assayed for Ang II by specific ELISA (Bachem America, Torrence, CA) according to the recommended number III protocol^13,14,20,43^. Urines were also assayed for levels of albumin and creatinine (ELISA, Albuwell and Creatinine Companion, Exocell, Inc., Philadelphia, PA)^13,14^ and glucose (Glucose colorimetric kit, Cayman Chemical, Ann Arbor, MI).

For tissue studies, mice were euthanized at the age of 8 or 24 weeks. Blood samples were collected by cardiac puncture. The kidneys were isolated, decapsulated and weighed. The left kidneys were processed for histology and immunostaining, and the right kidneys were used for isolation of renal proximal tubules (RPTs) by Percoll gradient^13,14,20,42,43^. Aliquots of freshly-isolated RPTs from individual animals were used immediately for total RNA isolation and Western blotting.

### Serum and urine biochemical measurements

Serum and urine sodium, phosphorus and calcium were measured by the Comparative Medicine and Animal Resources Centre, McGill University (Montreal, QC, Canada).

### Glomerular filtration rate

The glomerular filtration rate (GFR) was estimated with fluorescein isothiocyanate inulin as recommended by the Animal Models of Diabetic Complications Consortium (http://www.diacomp.org/) with slight modifications^13,14^.

### Intraperitoneal glucose tolerance Test

An intraperitoneal glucose tolerance test (IPGTT) was performed after 6 h fasting in non-anesthetized mice at the age of 23 weeks, as described previously^44^.

### Real time-quantitative polymerase chain reaction

Real time-quantitative polymerase chain reaction (RT-qPCR) analyses were performed to quantify the relative expression of *Hnrnpf*, *Agt*, *Sglt2*, *fibronectin I* (*FN1*) and *ribosomal protein L13A* (*RPL13A*) in isolated RPTs as described previously^13,14,20,43^ with specific primers (Supplemental Table 1).

### Western blotting

Western Blotting (WB) was performed in isolated RPTs as described previously^13,14,20,43^. Details of the sources of antibodies and working dilutions are listed in Supplemental Table 2.

### Histology

Kidney sections were stained with periodic acid Schiff (PAS) as previously described^13,14,20,43^. Masson’s Trichrome staining, Sirius Red staining and immunostaining for Fn1 were performed to assess tubule-interstitial fibrosis. Semi-quantitation of the relative staining was done by NIH Image J software (http://rsb.info.nih.gov/ij/). Mean glomerular tuft and RPTC volumes, and the tubular luminal area were determined by the methods of Weibel^45^ and Gundersen^46^, as described previously^47,48^.

### Immunofluorescence staining

Immunofluorescence (IF) staining was performed on 3-μm tissue sections from mouse kidney fixed in formalin and embedded in paraffin followed by staining with ALEXA FLUOR-594-labeled secondary antibody (Invitrogen). Proximal tubules were identified by fluorescein-labeled lotus tetragonolobus lectin (LTL, a marker of renal proximal tubule^49^) (Vector Labs, Burlingame, CA). Image quantification and merge were assessed by ImageJ software (http://rsb.info.nih.gov/ij/). To quantify the amount of Sglt2 expression, the pixel intensity of Sglt2 was divided by LTL intensity. To calculate the average ratio, 6 sections per mouse, 6 mice per group were analyzed.

### Human renal proximal tubular cells with or without *HNRNPF*

Human renal proximal tubular cells (RPTCs) (HK-2) cells are derived from a normal adult male human kidney transfected with the human papilloma virus 16 (HPV-16) E6/E7 genes^50^. KO of HNRNPF in HK-2 was performed by the CRISPR-Cas9 genome editing method provided by Invitrogen (TrueGuide™). Briefly, the day before transfection, HK-2 cells (2.5 × 10^5^ cells per well) were cultured in a 1:1 mixture of DMEM and Ham’s F12 medium containing 10% of FBS in 6-well plate. OPTI-MEM medium with Lipofectamine Cas9 Plus™ Reagent (Cat. No. CMAX00001, Invitrogen) and the mixture of 37.5 pmol TruCut™ Cas9 Protein v2 (Cat. No. A36497, Invitrogen) and 37.5 pmol gRNA (crRNA (Cat. No. A35509, CRISPR1099776_CR, Invitrogen):tracrRNA (Cat. No. A35506, Invitrogen)) were transfected to HK-2 and cultured for 2 days at 37 °C. Single cell clones were then isolated by using limiting dilution cloning in 96-well plates. The positive clones were identified for the absence of HNRNPF by WB of cellular extracts and confirmed by PCR of genomic sequence. The clones with HNRNPF expression were used as controls.

To test the pharmacologic effect of SGLT2 inhibition on SGLT2 and AGT expression, HK-2 cells were harvested after 24 hours of culture in serum-free normal glucose (5 mM) DMEM in the absence or presence of 0.5 mM canagliflozin as described by Pirklbauer *et al*.^51^. WB and RT-qPCR were used to quantify SGLT2 and AGT protein and mRNA expression, respectively.

### Canagliflozin treatment in wild-type (WT) mice

To investigate the impact of Sglt2 inhibition and *Agt* expression in RPTCs *in vivo*, male and female WT mice were treated with or without canagliflozin (0.2 mg/ml in drinking water) at the age of 4 weeks as described previously^52^. Body weight, blood and urinary glucose and SBP were monitored weekly. The mice were euthanized at the age of 8 weeks. The left kidneys were processed for histology and immunostaining, and the right kidneys were used for isolation of RPTs and were used immediately for total protein and RNA isolation to quantify protein and mRNA expression of Agt and Sglt2 by WB and RT-qPCR, respectively.

### Statistical analysis

The data are expressed as means ± SEM. Statistical significance between the experimental groups was analyzed by Student’s *t*-test or 1-way ANOVA (analysis of variance) and the Bonferroni test as appropriate. p < 0.05 values were considered to be statistically significant.

## Supplementary information

Supplementary Figures And Tables
