# Supplementary material for: Tubular Deficiency of Heterogeneous Nuclear Ribonucleoprotein F Elevates Systolic Blood Pressure and Induces Glycosuria in Mice
Source: Sci Rep. 2019 Oct 31;9:15765. doi: 10.1038/s41598-019-52323-1 (PMC6823451; doi:10.1038/s41598-019-52323-1)
Supplement: Supplementary file 1 — Supplementary Figures And Tables [file 41598_2019_52323_MOESM1_ESM.pdf]

Supplementary Tables and Figures

Tubular Deficiency of Heterogeneous Nuclear Ribonucleoprotein F Elevates Systolic Blood Pressure and Induces Glycosuria in Mice

Chao-Sheng Lo[1], Kana N. Miyata[1], Shuiling Zhao[1], Anindya Ghosh[1], Shiao-Ying Chang[1], Isabelle Chenier[1], Janos G. Filep[2], Julie R. Ingelfinger[3], Shao-Ling Zhang[1]*, John S.D. Chan[1]*

Supplemental Table 1 (Primers)

Gene	Primer sequences	Species	Reference Sequence
Recombinase (Cre)	S:CCTGGAAAATGCTTCTGTCCG AS:CAGGGTGTTATAAGCAATCCC		NC_005856.1 (Genotyping)
Floxed/wild type Hnrnpf	S:ACCTGCCAAATACCCATGCT AS:CAGATTGGACCGAACTCCCC	Mouse	NC_000072.6 (Genotyping)
Hnrnpf	S:CTCTCCGACTGCACAATTCAT AS:TCCATCTCGGTTCTGTGTGAC	Mouse	NM_133834.2
Angiotensinogen (Agt)	S: CCACGCTCTCTGGATTTATC AS: ACAGACACCGAGATGCTGTT	Mouse	NM_007428.3
Angiotensinogen (AGT)	S: AACTGGTGCTGCAAGGATCT AS: TCTCTCTCATCCGCTTCAAG	Human	NM_000029.4
Sglt2(Slc5a2)	S: TTGGTGTTGGCTTGTGGTCTAT AS: ATGTTGCTGGCGAACAGAGA	Mouse	NM_133254.4
SGLT2(SLC5A2)	S: GACACGGTACAGACCTTCGTCAT AS: CTCCCAGGTATTTGTCGAAGAGA	Human	NM_003041.4
Rpl13a	S:GCCCCACAAGACCAAGAGAG AS:TAGGCTTCAGCCGAACAACC	Mouse	NM_009438.5
RPL13A	S: GCCCTACGACAAGAAAAAGCG AS: TACTTCCAGCCAACCTCGTGA	Human	NM_012423.4
FN1	S:GGCCTGAACCAGCCTACAG AS:TGAGCTTAAAGCCAGCGTCA	Mouse	NM_010233.2
Sglt1(Slc5a1)	S:GGTGACGATGCCGGAGTATC AS：AGGCCCAAGGCTAGATTGATG	Mouse	NM_019810.4 (Supplemental Figures 2c)
SGLT2 (for HK2)	S: TTCGGGTCTCTTCGACAAAT AS：GCGTGTAGATGTCCATGGTG	Human	(Supplemental Figures 6b)
5(XhoI)SGLT2 prom-1986	S:TTAACTCGAGGTCTGTAACACACACGTG TC	Human	Clone promoter
3(BglII)SGLT2 prom+22	AS：AATTAGATCTCATTCTCCCCAGGATCTGC	Human	Clone promoter

Supplemental Table 2 (Antibodies)

Antibody	Host	Dilution for WB	Dilution for IHC	Dilution for IF	Company
Hnrnpf	Rabbit polyclonal	1:2000			Specifically recognizing Hnrnpf (CTARRYIGIVKQAGLER) were generated in our laboratory (1)
Hnrnpf	Rabbit polyclonal			1:100	Abcam (ab50982)
Sglt2 (D-6)	Mouse monoclonal	1:100			Santa Cruz (sc-393350)
Sglt2	Rabbit polyclonal			1 :200	Abcam (AB85626)
Agt	Rabbit polyclonal	1:2000	1:200		Specifically recognizing Agt were generated in our laboratory(2)
β-Actin	Mouse Monoclonal	1 :20000			Sigma-Aldrich (A5441)
FLUORESCEIN LABELED LOTUS TETRAGONOLOBUS LECTIN (LTL)				1:200	Vector Labs (FL-1321)
Fibronectin	Rabbit polyclonal		1:200		Sigma-Aldrich (F3648)

(1)Wei CC, Guo DF, Zhang SL et al. Heterogeneous nuclear ribonucleoprotein F modulates angiotensinogen gene expression in rat kidney proximal tubular cells. J Am Soc Nephrol, 16:616–628, 2005.
(2)Wang L, Lei C, Zhang SL et al. Synergistic effect of dexamethasone and isoproterenol on the expression of angiotensinogen in immortalized rat proximal tubular cells. Kidney international, 53: 287-295, 1998.

Supplemental Figures

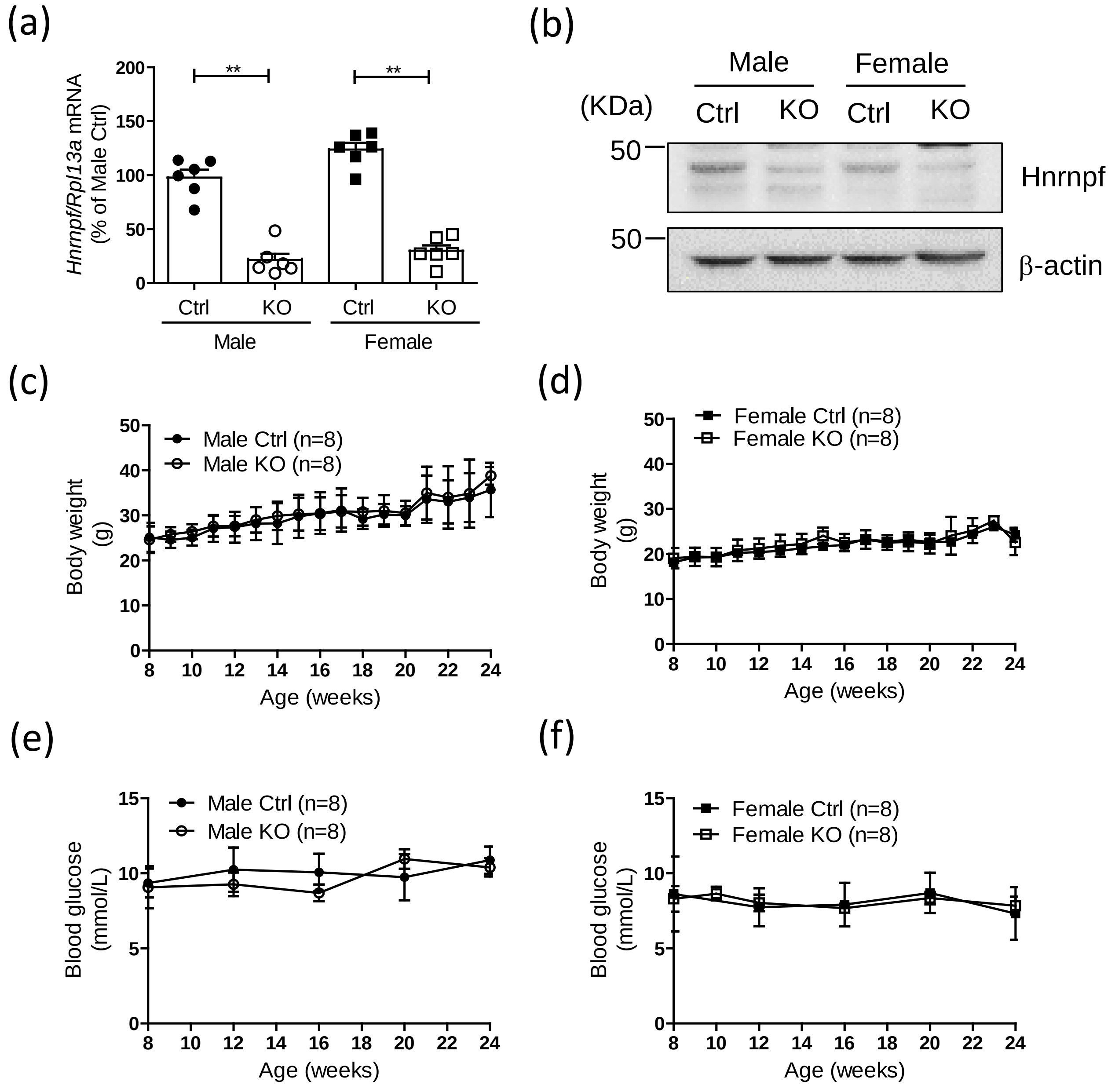

Supplemental Figure 1 Hnrnpf mRNA (a) and protein (b) expression in RPTs freshly isolated from male and female Ctrl and KO at the age of 8 weeks. Renal tubular Hnrnpf deficiency did not influence body weight gain (c and d) nor non-fasting blood glucose level (e and f) in male and female mice from age of 6 to 24 weeks. **P<0.01, KO versus Ctrl; n=6 per group.

Supplemental Figures

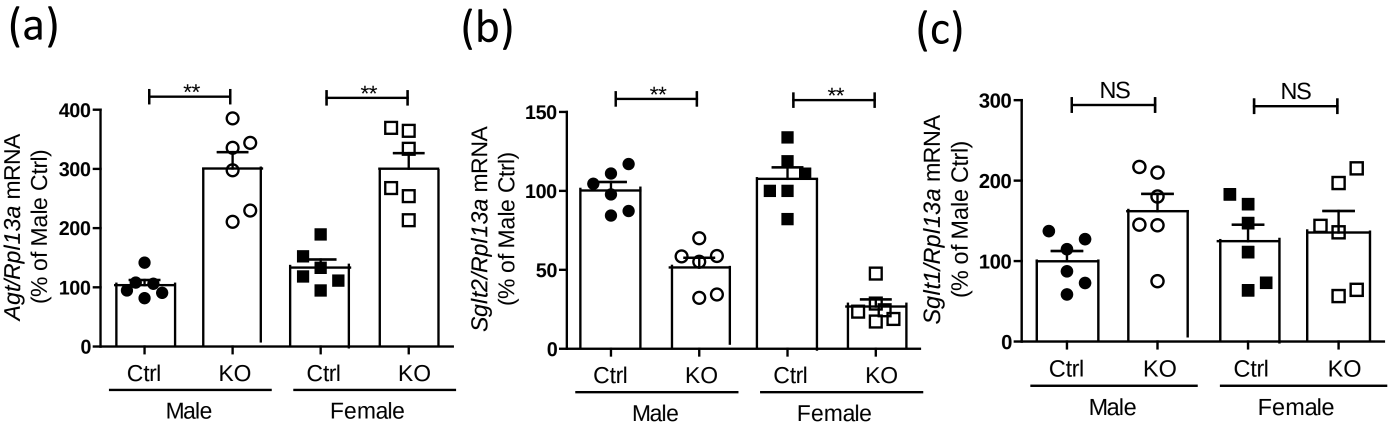

Supplemental Figure 2 (a) RT-qPCR revealed significant increases of *Agt (a)* and decreases of *Sglt2 (b)* expression in RPTs isolated from male and female KO mice at 8 weeks of age. No change of *Sglt1 (c)* expression was observed in RPTs from male and female KO mice at 24 weeks of age. **P < 0.01. KO versus Ctrl. NS, not significant; n=6 per group.

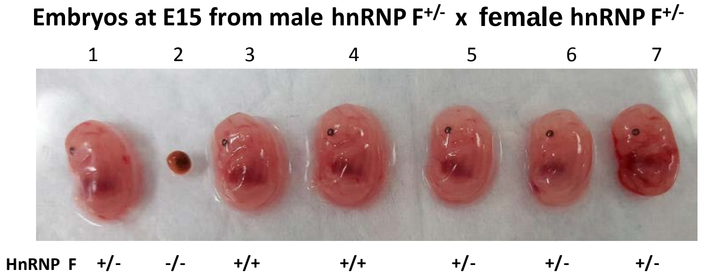

Supplemental Figure 3. Day 15 embryos from heterozygous CMV-hnRNP $F^{+/-}$ parents. One resorbed embryo with global hnRNP $F^{-/-}$ KO (#2), 4 embryos with heterozygous hnRNP $F^{+/-}$ (#1, #5, #6 #7) and 2 wild type (WT) embryo (hnRNP $F^{+/+}$) (#3, #4) were observed. Similar results were observed in several other litters with 1 to 3 resorbed embryos per litter.

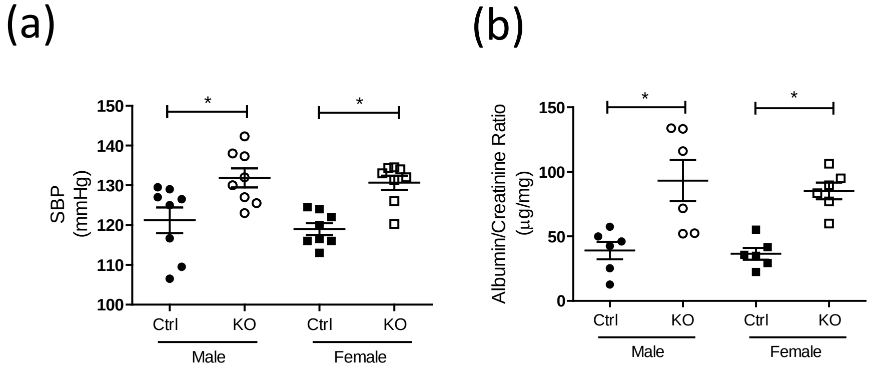

Supplemental Figure 4. (a)Systolic blood pressure (SBP) (n=8 mice; 8 weeks old), (b) Urinary albumin/creatinine ratio (ACR) (n=6 mice; 8 weeks old). Values are means ± SEM, *P < 0.05, KO versus Ctrl.

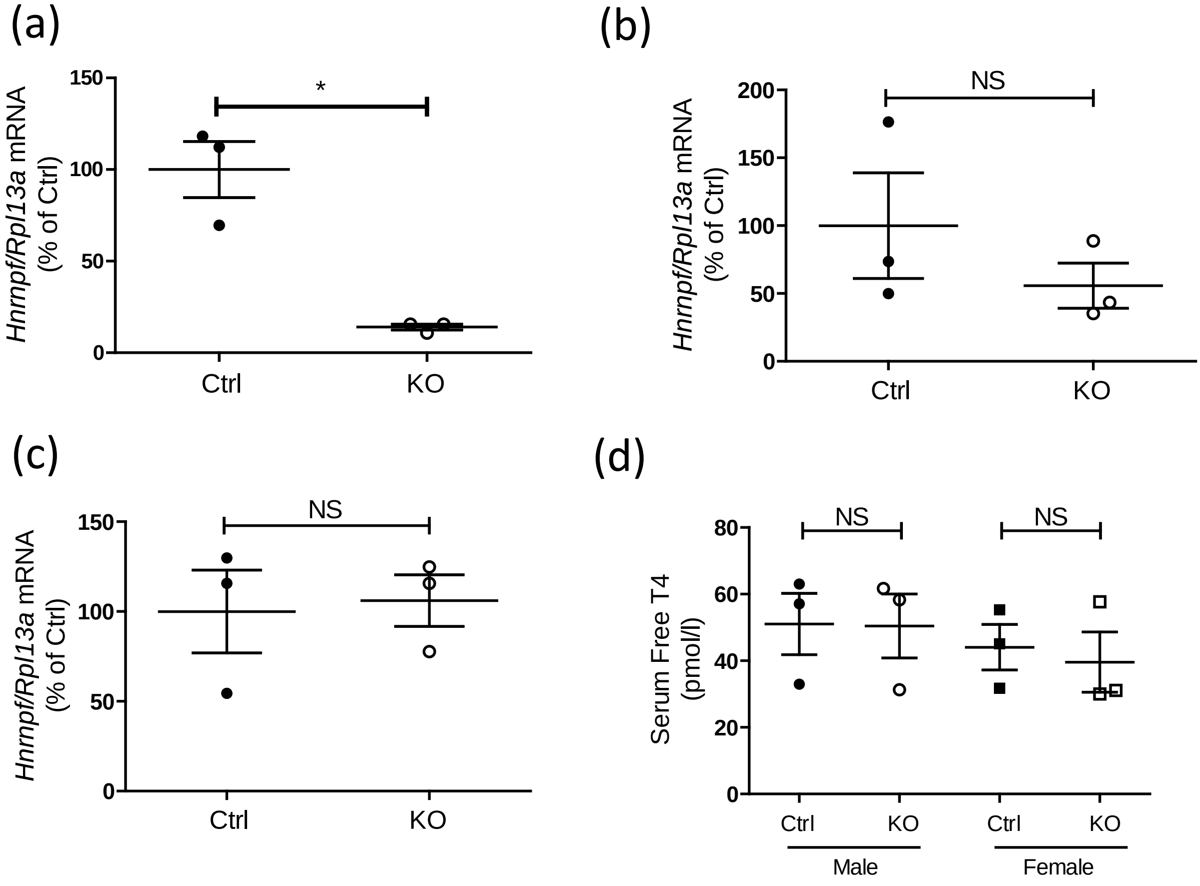

Supplemental Figure 5. RT-qPCR of hnRNP F mRNA levels in (a) Kidney, (b) Thyroid gland, (c) Hindbrain of Control and Pax8-HnRNP F KO mice. (d) Serum T4 levels in Control and Pax8-HnRNP F KO mice measured by ELISA. N=3. *P < 0.05, KO versus Ctrl. NS, not significant.

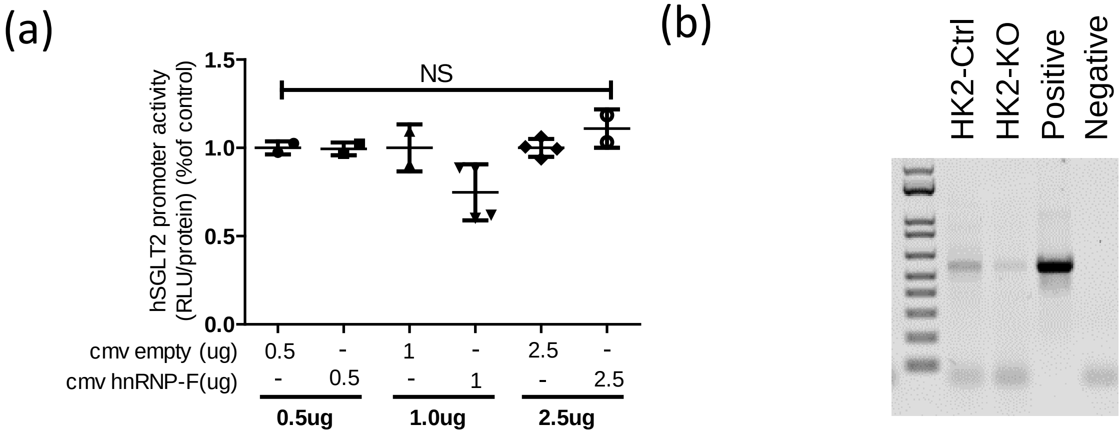

Supplemental Figure 6. (a) Transfection of HNRNPF cDNA had no effect on SGLT2 promoter activity. (b) Similar molecular size of SGLT2 was found in HK-2 and HK-2-HNRNPF KO. N=2 independent experiments. NS, not significant.
